# The Impact of Domestic Cooking Methods on Myrosinase Stability, Glucosinolates and Their Hydrolysis Products in Different Cabbage (*Brassica oleracea*) Accessions

**DOI:** 10.3390/foods10122908

**Published:** 2021-11-24

**Authors:** Omobolanle O. Oloyede, Carol Wagstaff, Lisa Methven

**Affiliations:** Department of Food and Nutritional Sciences, Harry Nursten Building, University of Reading, Whiteknights, Reading RG6 6DZ, UK; c.wagstaff@reading.ac.uk (C.W.); l.methven@reading.ac.uk (L.M.)

**Keywords:** *Brassica oleracea*, cabbage, myrosinase stability: glucosinolates, glucosinolate hydrolysis products, isothiocyanates, epithionitriles, steaming, microwaving, stir-frying

## Abstract

Glucosinolate hydrolysis products are responsible for the health-promoting properties of *Brassica* vegetables. The impact of domestic cooking on the myrosinase stability, glucosinolates and hydrolysis products in 18 cabbage accession was investigated. Cabbages were steamed, microwaved, and stir-fried before analysis. Cooking significantly affected myrosinase stability and glucosinolate concentrations within and between cabbage morphotypes. Myrosinase was most stable after stir-frying, with up to 65% residual activity. Steaming and microwaving resulted in over 90% loss of myrosinase activity in some accessions. Stir-frying resulted in the greatest decrease in glucosinolate concentration, resulting in up to 70% loss. Steamed cabbages retained the highest glucosinolates after cooking (up to 97%). The profile and abundance of glucosinolate hydrolysis products detected varied across all cooking methods studied. Cooking reduced the amounts of nitriles and epithionitriles formed compared to raw samples. Steaming led to a significant increase in the concentration of beneficial isothiocyanates present in the cabbage and a significantly lower level of nitriles compared to other samples. Microwaving led to a reduction in the concentrations of both nitriles and isothiocyanates when compared to other cooking methods and raw cabbage. The results obtained help provide information on the optimal cooking methods for cabbage, suggesting that steaming may be the best approach to maximising beneficial isothiocyanate production.

## 1. Introduction

Consumption of *Brassica* or cruciferous vegetables such as cabbage (*Brassica oleracea*) is reported to result in chemo-protective effects [1]. This has been attributed to the high amounts of glucosinolates (GSLs) they contain. When plant tissue is damaged as a result of autolysis, plant injury, processing or chewing, GSLs are exposed to, and hydrolysed by, endogenous myrosinase. Upon hydrolysis, glucose and an unstable aglycone (thiohydroxamate-*O*-sulfonate) are produced. The unstable aglycone (thiohydroxamate-*O*-sulfonate) immediately rearranges to form different hydrolysis products such as isothiocyanates (ITCs), thiocyanates, nitriles, epithionitriles (EPTs), and oxazolidine-2-thiones, depending on the conditions of the reaction [2]. Nitriles and EPTs are formed in the presence of epithiospecifier proteins (ESP) instead of the more beneficial ITCs [3]. ITCs and indoles commonly found in cabbages such as sulforaphane (SFP), erucin (ER), allyl ITC (AITC), 2-phenyethyl ITC (PEITC), iberin (IB) and indole-3-carbinol (I3C) are reported to be responsible for some of the health-promoting properties of *Brassicas* [2]. SFP, the most studied of all the ITCs, is reported to possess chemoprotective, antioxidative, antimicrobial and neuroprotective properties [4,5,6,7,8,9]. AITC has been found to be potent against bladder [10,11], breast [12] and lung [13] cancer cells. I3C is also known to have anti-cancerous activities on reproductive organs, reducing the proliferation of cancer cells in the breast, prostrate, cervical and colon cell lines and preventing tumour development in rodents [14,15,16]. GSLs and glucosinolate hydrolysis products (GHPs) are also partly responsible for the bitter taste and pungent flavour and aroma of *Brassica* vegetables, which can reduce consumer acceptability of *Brassicas* [17,18]. Cox et al. [19] reported that *Brassica* acceptance was low in adults due to their sensory characteristics.

Where GSL hydrolysis does not occur in the process of preparing *Brassica* for consumption, it can occur as a result of microbial activities in the gastrointestinal tract of humans. However, despite the ability of microorganisms in the human gut to hydrolyse glucosinolates, it has been reported that the conversion is at least three times less efficient when compared to glucosinolate hydrolysis by myrosinase [20]. In a recent in vivo study conducted by Okunade et al. [21], the addition of exogenous myrosinase from brown mustard powder to cooked and pureed broccoli where myrosinase had been inactivated resulted in a four times increase in sulforaphane bioavailability compared to when the pureed broccoli was consumed alone. It is, therefore, important to ensure that myrosinase enzyme remains active during the consumption of *Brassica* vegetables.

Cabbages, like other *Brassica* vegetables, are mostly subjected to some form of thermal processing or domestic cooking before consumption. Cabbages are commonly boiled, steamed, stir-fried, or microwaved prior to consumption. Thermal cooking processes are considered one of the most important factors affecting the stability of myrosinase enzyme and ESP stability and the profiles, concentrations, and bioavailability of GSLs and their hydrolysis products [22]. This, in turn, can influence the health benefits that can be derived from the consumption of these vegetables making it crucial to determine the effect of cooking processes on myrosinase stability, GSLs and GHP formation within plant tissues.

Cooking cabbage can result in total or partial ESP and myrosinase inactivation, which, in turn, influences the type of GHPs formed. The time and temperature of cooking, vegetable matrix and degree of tissue damage all influence the changes observed during cooking [23]. Several studies have shown that myrosinase is inactivated during the thermal processing or domestic cooking of cabbage, leading to a decreased production of beneficial hydrolytic compounds [24,25,26]. Most of these studies, however, were based on crude myrosinase extracts or cabbage juice [24,26,27,28]. Myrosinase enzyme, when present within plant tissue, has been shown to have greater thermal stability than its crude extract, with this stability attributed to the rate at which the core temperature increased [24].

Previous studies on GSL concentrations in cooked cabbage showed conflicting results. Some authors have reported an increase in GSL content after microwaving cabbage [24,28]. Rungapamestry et al. [25], Song and Thornalley [29] and Xu et al. [30] reported minimal losses or no change in GSL concentration after steaming and microwaving cabbage. Xu et al. [30] recorded a 77% loss in GSL concentration after stir-frying. GSL losses during cooking have mostly been linked to leaching into cooking water [24,31]. The variation in myrosinase and GSL stability after processing can be attributed to different cooking conditions and the size of cut cabbage pieces, which, in most cases, do not represent standard domestic ways of cooking cabbage. Some of these studies processed the cabbages under much longer time–temperature combinations compared to what would normally be applicable during the domestic cooking of cabbage [24,26]. Furthermore, most of these studies [24,26,28] have focused on closed heart cabbages (mostly red and white cabbage) with limited data available on open leaf varieties such as black kale. There is insufficient evidence to date linking high myrosinase activity and/or GSL accumulation to high stability after processing/cooking; hence, studies considering both the stability of myrosinase and GSLs within plant tissue for various *B. oleracea* species are needed. Available studies have focused on the effect of cooking on specific GSLs and their GHPs, or just ITCs [25,29,32,33,34,35,36,37]. Some of these studies have been conducted in model systems [36], which do not consider the various reactions occurring within the plant matrix that can influence the GSL–myrosinase system during domestic cooking processes.

In our earlier paper investigating the effect of accession identity and growing conditions on myrosinase activity, GSL and GHP in 18 cabbage accessions, we discussed in detail the results of myrosinase activity, as well as the profile and concentration of GSL and GHP, between the raw accessions [38]. In this paper, the focus is on the effects of cooking on these accessions. This study, therefore, examines the effect of steaming, microwaving, and stir-frying on myrosinase stability, GSL and GHP profiles and concentrations in 18 cabbage accessions. Cooking times were chosen to represent standard domestic practices. It was hypothesised that, through controlled domestic cooking processes, myrosinase and GSL stability would be maximised, thereby increasing the production of ITCs, and improving the health benefits associated with cabbage consumption. It was also hypothesised that the genetic background of the cabbage and its morphotype would impact the observed stability of myrosinase and GSLs, and the production of GHPs.

## 2. Materials and Methods

### 2.1. Plant Material

The seeds of the cabbage accessions used for this study were sourced from the University of Warwick Crop Centre Genetic Resources Unit (Wellesbourne, UK), sown in controlled environments, transplanted to pots (2.5 L) and left to grow in the glasshouse for a short time before transplanting to 7 metre beds on the field. A detailed cultivation protocol can be found in Oloyede et al. [38]. Eighteen cabbage accessions from six different cabbage morphotypes (black kale (BK), wild (WD), tronchuda (TC), savoy (SC), red (RC), and white (WC)) were selected based on their head formation (closed heart or open leaf), geographical location and whether they were of hybrid descent (Supplementary Table S1). Cabbages were grown between 7th March and 25th November in the plant growth facilities, Whiteknights campus of the University of Reading, UK. White cabbage accession WC3, did not germinate under the growing condition.

Upon reaching commercial maturity (based on visual inspection), cabbages were harvested, immediately placed on ice in freezer bags and stored in a cold room (4 °C) for 24 h prior to processing. Average weight of each cabbage head per plant was 700 g and 300 g for closed heart and open leaf, respectively. For detailed climatic data and cross section of cultivated cabbages, refer to Supplementary Table S1 in this paper and Figures S1 and S2 in our earlier paper, Oloyede et al. [38].

### 2.2. Reagents and Chemicals

Sinigrin standard was obtained from Santa Cruz Biotechnology (Heidelberg, Germany) and D-glucose determination kit from R-Biopharm Rhone (Heidelberg, Germany). All other chemicals used were purchased from Sigma–Aldrich (Dorset, UK).

### 2.3. Cabbage Thermal Processing

In order to achieve a representative sample and remove senescent leaves, the outer leaves and central core of 4–5 cabbage heads (biological replicates) were removed and discarded. Cabbages were chopped into pieces of approximately 1 cm in width using a kitchen knife (representing how cabbages would normally be prepared by consumers), mixed together, washed under running tap water, and drained of excess water using a salad spinner (OXO Good Grips Clear Manual Salad Spinner, Chambersburg, PA, USA). Cabbages were subjected to steaming, microwave, or stir-fry cooking. Unprocessed (raw) cabbage samples were used as controls. Cooking methods were chosen to represent common ways of cooking cabbage.

Time and temperature combinations used for each method were based on a preliminary consumer study with 60 participants to determine consumer acceptability of the samples as steamed, microwaved, and stir-fried cabbage (Supplementary Table S2). These conditions were deemed acceptable with a mean score of between 2.7 and 3.8 on a 5-point degree of cooking scale, where “3” represents ‘just about right’ for extent of cooking (scale from not cooked enough “1”, too much too overcooked “5”).

#### 2.3.1. Steaming

The method of Rungapamestry et al. [25] was adopted with slight modifications. A total of 120 g cabbage was placed in the topmost layer of a 3-tier, 18 cm stainless steel steamer (Kitchen craft, Birmingham, UK) containing already-boiling water (in the lowest layer) and allowed to steam for 2 min. Core temperature of cabbage during steaming ranged between 75 and 80 °C and was measured using a temperature probe.

#### 2.3.2. Microwaving

The method of Rungapamestry et al. [25] was adopted. A total of 120 g of cabbage was put into 1-pint Pyrex glass jug, 16 mL water was added, and the jug was covered with a PVC cooking film pierced with 9 holes. Cabbages were microwaved for 3 min. Microwaving was carried out using a 900 W microwave at 60% power output (SANYO microwave oven EM-S355AW/AS, Osaka, Japan). A microwave thermometer was used to measure the core temperature of the cabbage during processing. Core temperature during processing ranged between 88 and 95 °C.

#### 2.3.3. Stir-Frying

Cabbage samples were stir-fried as described by Rungapamestry et al. [39] with modifications. A total of 120 g cabbage was stir-fried in a frying pan for 90 s in 5 mL of preheated olive oil (100 °C) (Asda, Reading, UK) with continuous stirring using a wooden spatula. Core temperature of cabbage during stir-frying ranged between 65 and 70 °C and was measured using a temperature probe.

Samples were put into sterilin tubes immediately after cooking, placed on ice and transferred to a −80 °C freezer. Frozen samples were freeze-dried (Stokes freeze drier, Philadelphia, PA, USA), ground using a tissue grinder (Thomas Wiley^®^ Mini-Mill, Thomas Scientific, Swedesboro, NJ, USA) and stored at −20 °C until further analysis.

### 2.4. Myrosinase Enzyme Extraction and Assay and Protein Content Analysis

Myrosinase enzyme was extracted using the method described by Ghawi et al. [32] with slight modifications, as described in our previous paper, Oloyede et al. [38]. Myrosinase enzyme was extracted from a 0.1 g sample (at 5 °C) using polyvinylpolypyrrolidone (PVPP) and Tris- HCL buffer. Using a D-glucose determination kit, myrosinase activity was determined following the coupled enzyme method as described by Gatfield and Sand [40] and Wilkinson et al. [41] with some modifications, as outlined in our preceding paper [38]. Myrosinase activity of the samples was calculated using a calibration curve prepared from myrosinase enzyme. One unit of myrosinase activity was defined as the amount of enzyme that produces 1 µmol of glucose per minute from sinigrin substrate at pH 7.5.

Protein content from the enzyme extract was determined using the Bradford method [42]. The method is based on protein complex formation with Brilliant Blue G dye with absorbance read 595 nm in a spectrophotometer (Perkin Elmer, Shelton, CT, USA). A standard curve was constructed using Bovine serum albumin (BSA) and used to calculate protein concentration in the enzyme extracts from which myrosinase-enzyme-specific activity (U/mg protein) was determined.

### 2.5. Glucosinolate and Glucosinolate Hydrolysis Products Analysis

GSLs and GHPs were extracted following the methods described by Bell et al. [43] and Bell et al. [44], respectively, with modifications as described in our earlier paper Oloyede et al. [38]. GSLs were extracted with 70% methanol, analysed by LC-MS/MS (Agilent, Bracknell, UK), and quantified using sinigrin hydrate standard. Six concentrations of sinigrin hydrate (14–438 µg/mL) were prepared with 70% methanol and used to prepare an external calibration curve (*r*^2^ = 0.996). Compounds were identified using their mass parent ion, characteristic ion fragments and through comparing with ion data from literature (Table 1).

GHPs were extracted using dichloromethane and analysed by GC-MS (Agilent, Manchester, UK). Compounds were identified using the literature on ion data (Table 2; see Supplementary Figure S1 for GC-MS chromatograms) and quantified based on an external standard calibration curve. Five concentrations (0.15–0.5 mg/mL) of sulforaphane standard (Sigma Aldrich, UK) were prepared in DCM (*r*^2^ = 0.99). Data analysis was performed using ChemStation for GC-MS (Agilent, Manchester, UK).

### 2.6. Statistical Analysis

Results are the average of three biological or processing replicates (each replicate consisting of leaves from 4–5 cabbage heads) and two technical replicates (*n* = 6). All statistical analyses were performed in XLSTAT (version 2019.4.2, Addinsoft, Paris, France). Data obtained were analysed using 2-way ANOVA with both cabbage accession (or morphotype) and processing conditions (raw, steamed, microwaved, and cooked) fitted as treatment effects. Tukey’s HSD multiple pairwise comparison test was used to determine significant differences (*p* < 0.05) between samples. Principal component analysis (PCA) and multifactor analysis (MFA) were used to visualise the data in a minimum number of dimensions (two or three). MFA makes it possible to simultaneously analyse several tables of variables, showing relationships and correlations between the observations and variables, which were analysed in such a way that tables that include more variables do not outweigh other tables in the analysis.

## 3. Results and Discussion

### 3.1. Effect of Domestic Cooking on Residual Myrosinase Enzyme Activity (Relative Activity) across Cabbage Morphotypes and Accessions

**Figure 1:**
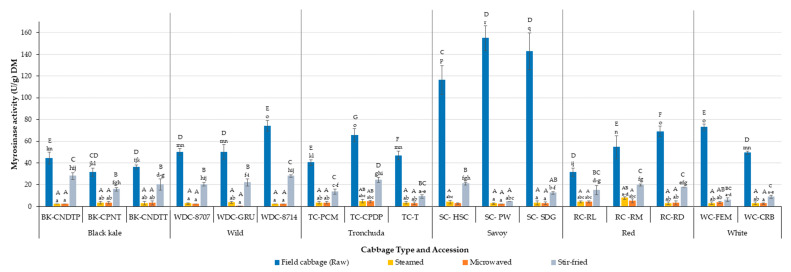
Comparison of the myrosinase activity of raw versus cooked cabbage morphotypes and accessions (U/g DW). Values are means of three biological (raw samples) or processing (cooked samples) replicates (each replicate comprising 4–5 cabbage heads) and two technical replicates (*n* = 6). Error bars represent standard deviation from mean values. Letters “A–G”: bars not sharing a common uppercase letter differ significantly (*p* < 0.0001) between accessions and treatments within a cabbage morphotype. Letters “a–r”: bars not sharing a common lowercase letter differ significantly (*p* < 0.0001) between cabbage morphotypes, accessions, and treatments. Key: BK-CNDTP: cavolo nero di toscana o senza palla; BK-CPNT: cavolo palmizio; BK-CNDTT: cavolo nero di toscana o senza testa; WD-8707: wild cabbage 8707; WD-GRU: wild cabbage 7338; WD-8714: wild cabbage 8714; TC-PCM: penca mistura; TC-CPDP: penca povoa; TC-T: tronchuda; SC-HSC: hybrid savoy wirosa; SC-PW: pointed winter; SC-SDG: dark green; RC-RL: red langendijker; RC-RM: rocco marner (hybrid); RC-RD: red Danish; WC-FEM: early market; WC-CRB: couve repolho.

The myrosinase stability of cabbage accessions after domestic cooking was studied and the results are presented in Figure 1, with relative activity results presented in Supplementary Table S3. Relative activity is defined as the ratio of myrosinase activity of processed (cooked) cabbage to unprocessed (raw) cabbage (A/A_0_). Domestic cooking affected the stability of myrosinase enzyme. Myrosinase stability differed significantly (*p* < 0.05) between domestic cooking processes, where there was no difference between steaming and microwaving (*p* = 0.912), but these processes both differed significantly from stir-frying (*p* < 0.0001). Myrosinase was most stable after stir-frying, retaining up to 65% (i.e., A/A_0_ = 0.65, Supplementary Table S3) of its activity in some studied accessions. Steaming and microwaving resulted in losses of myrosinase activity of up to 98% and 99%, respectively, with the highest stability of 15% and 13%, respectively. Rungapamestry et al. [39], in their study of broccoli florets, reported that stir-frying retained the highest myrosinase activity (17%) compared to boiling (14%).

The effect of domestic cooking processes on myrosinase stability varied among cabbage morphotypes and accessions and will be discussed in more detail later. The stability of myrosinase in different *Brassica* vegetables under different processing conditions has been discussed by several authors [24,25,26,27,39,55]. Differences in myrosinase stability as a result of cooking can be attributed to the maximum core temperature of the vegetable during heating. In our study, stir-frying had the lowest core temperature (65–70 °C) compared to steaming (75–80 °C) and microwaving (88–95 °C). It has previously been reported that, to prevent myrosinase inactivation, the maximum core temperature that cabbage should reach is between 50 and 60 °C, which can be achieved by steaming for 7 min or microwaving (700 W) for 120 s [25]. However, in the study stated, the cabbage samples were cut into wedges, which is not representative of how cabbages are generally prepared before cooking, so the cooking times to reach the same core temperature in cabbage that is more finely chopped would be shorter.

Verkerk and Dekker [24] reported that inactivation of myrosinase enzyme during microwave cooking is affected by the time–energy output combination. Their study showed that a considerable amount of myrosinase activity was retained when red cabbage was microwaved at 180 W for 24 min and 540 W for 8 min, while microwaving for 4.8 min at 900 W resulted in total loss of myrosinase activity even though the total energy output of all three processes was the same (259.2 KJ). The authors explained the resulting effect as a function of the time it takes for the cabbage to reach its maximum core temperature, with a higher energy output and shorter time reaching a high (100 °C) core temperature faster and maintaining that core temperature for the remaining cooking time, while the lower energy output with a longer cooking time resulted in a maximum core temperature of 90 °C at a much slower rate.

In the current study, a physical examination of the cooked cabbage samples showed that the stir-fried cabbage looked firmer than the steamed and microwaved cabbage, which can be a helpful way to assess the severity of the thermal process. The intense heat during stir-frying can lead to drying out of the surface area, thereby resulting in a firmer texture, which reduces the rate of heat penetration as a result of less damage to the cell wall [39,56].

### 3.2. Comparison of the Myrosinase Activity of Raw versus Cooked Cabbage Morphotypes and Accessions

Figure 1 shows the myrosinase activity and subsequent thermal stability of the 17 studied cabbage accessions. Significant differences (*p* < 0.0001) were observed in the myrosinase activity and stability of cabbages as a result of cabbage morphotype, accession, cooking method, and the interactions between these parameters.

There was no relationship between myrosinase activity in raw cabbage and myrosinase stability post-cooking; indeed, some accessions which had high activity in raw cabbage had the lowest stability. Raw savoy cabbage accessions (SC-HSC, SC-PW, and SC-SDG) had the highest myrosinase activity in all studied accessions (116.3, 142.6 and 154.8 U/g DW, respectively) while raw black kale accessions (BK-CNDTP, BK-CPNT, and BK-CNDTT) had the lowest myrosinase activity (31.5, 36.3 and 44.4 U/g DW, respectively). However, black kale, Tronchuda and red cabbage accessions had the highest enzyme stability, while savoy and white cabbage accessions, which had the highest myrosinase activity, were the least stable after domestic processing (Figure 1). As discussed earlier, steaming and microwaving resulted in lower myrosinase stability overall, with up to 99% inactivation occurring in some cases. However, a critical look at the stability of myrosinase in steamed and microwaved cabbages (Figure 1) shows that some accessions had relatively higher myrosinase stability compared to others. Red cabbage accessions RC-RM and RC-RL were the most stable, retaining up to 15% after steaming (RC-RM) and 13% after microwaving (RC-RL). This result is in agreement with the results of Yen and Wei [27], who stated that red cabbage myrosinase was more stable than white cabbage myrosinase after thermal processing.

A possible reason for the difference in myrosinase stability across accessions might be due to differences in the myrosinase isoenzymes found in each accession, with the red cabbage accessions having more thermally stable myrosinase isoenzyme. Red cabbage contains anthocyanins, which, in addition to being bioactive compounds with health promoting properties, are also pigments that offer effective protection to plants under stress [57,58]. Therefore, red cabbage is more adapted to stressful conditions, and it stands to reason that the myrosinase isoenzyme in red cabbage may also be adapted to operating under heat stress.

Different types of myrosinase isoenzymes have been identified and they vary between *Brassica* vegetables. They can differ to some extent in characteristics and activity, with distribution in plants appearing to be both organ- and species-specific [27,59]. Rask et al. [60] reported that some of the myrosinase isoforms form complexes by interacting with myrosinase-binding proteins, which may enhance their stability during processing.

The myrosinase activity values obtained in this study were higher in most cases than those reported by other authors [25,61], except in the case of white cabbage accessions, where values were similar to those obtained by Penas et al. [62]. This might be because, in most previous studies, cabbages were obtained from supermarkets, while in this study and the study conducted by Penas et al. [62], the cabbages were grown for the experiment and transferred into cold conditions immediately after harvest. These minimal transfer and storage times reduce the postharvest effects experienced by the supermarket samples.

There was no relationship found between accession origin, physical characteristics (open-leaf or heart-forming) and whether cabbages were hybrid on the myrosinase activity and stability of the accessions studied.

### 3.3. Protein Content and Specific Activity of Raw and Cooked Cabbages

The protein content and specific activity of cabbage myrosinase before and after cooking is presented in Table 3. There were significant (*p* < 0.05) differences in the protein content and specific activity of all accessions for both raw and cooked samples. Protein content decreased with cooking, with the rate of reduction corresponding to the severity of the cooking process. Stir-fried samples had significantly higher protein contents than steamed and microwave samples. Black kale and red accessions, with the highest protein content, also retained the most protein after stir-frying (up to 87% in BK-CNDTP) but the lowest after steaming and microwaving (up to 67% in steamed BK-CPNT). This can be attributed to the denaturation of protein into free amino acids during cooking.

Cooking led to a significant reduction (*p* < 0.05) in the specific activity of cabbage samples. The specific activity of the cabbages followed a similar trend to myrosinase activity and protein content, where specific myrosinase activity decreased with the severity of the cooking method. The result shows a correlation between myrosinase activity and specific activity, implying that denaturation of the protein is equal to denaturation of the enzyme. Stir-fried cabbages had the most stable specific activity and differed significantly (*p* < 0.001) from steamed and microwaved samples between accessions for all studied morphotypes, with the exception of savoy morphotype, where no significant difference was observed in specific activity between accessions for the studied cooking methods (Table 3). It is worth mentioning that the results obtained for savoy accessions were mostly due to the significantly higher specific activity of the raw samples, instead of a comparable stability across the cooking methods. Similar to our earlier discussion on myrosinase stability, samples with the highest specific activity were not always the most stable after cooking. For example, Savoy cabbage accessions (SC-HSC, SC-PW, and SC-SDG), which had the highest specific activity (4.7, 6.4 and 5.8 U/mg soluble protein, respectively) had the lowest stability after cooking, with an up 97% loss in specific activity after steaming and microwave cooking observed in SC-PW accession. On the other hand, black kale accessions, with some of the lowest specific activity in raw samples, retained the most specific activity after cooking, with up to 80% specific activity observed in BK-CNDTT accession. As expected, the result obtained is in agreement with myrosinase activity results discussed earlier, where black kale accessions with the least myrosinase activity were the most stable after domestic cooking. The differences observed in specific activity can be attributed to variations in myrosinase isoenzyme stability for the different morphotypes and accessions, as discussed in Section 3.1 and Section 3.2.

### 3.4. Effect of Domestic Cooking on GSL Profile and Concentration of Cabbage Accessions

**Figure 2:**
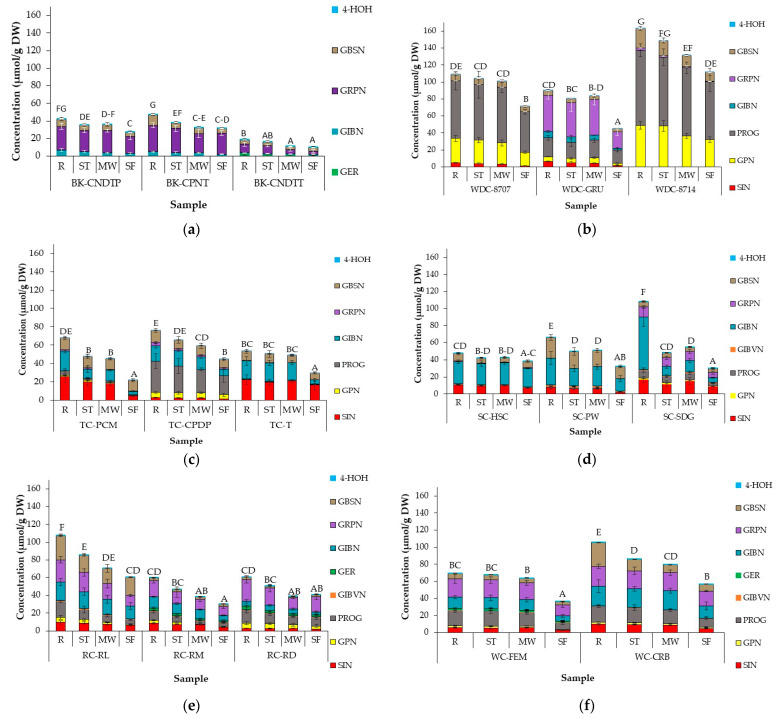
Glucosinolate concentrations (µmol/g DW) in different accessions of (**a**) Black kale; (**b**) Wild cabbage (**c**) Tronchuda cabbage; (**d**) Savoy cabbage; (**e**) Red cabbage and (**f**) White cabbage before and after domestic processing. Error bars represent standard deviation from mean values. Letters above bars refer to differences in total GSL concentration. Letters ‘A–G’: bars not sharing a common uppercase letter differ significantly (*p* < 0.05) between accession and cooking conditions within a cabbage morphotype (i.e., within each separate graph). For significant differences between cabbage morphotypes, accessions, and cooking methods (i.e., across the separate cabbage morphotype graphs) see Supplementary Table S4. Abbreviations: R = raw, ST = steamed; MW = microwaved; SF = stir-fried; BK-CPNT: cavolo palmizio; BK-CNDTT: cavolo nero di toscana o senza testa; WD-8707: wild cabbage 8707; WD-GRU: wild cabbage 7338; WD-8714: wild cabbage 8714; TC-PCM: penca mistura; TC-CPDP: penca povoa; TC-T: tronchuda; SC-HSC: hybrid savoy wirosa; SC-PW: pointed winter; SC-SDG: dark green; RC-RL: red langendijker; RC-RM: rocco marner (Hybrid); RC-RD: red Danish; WC-FEM: early market; WC-CRB: couve repolho. For abbreviations of compounds, see Table 1 (GSLs).

GSL profile and concentrations for all samples before and after cooking are presented in Figure 2, with significant differences within and between cabbage morphotypes presented in Supplementary Table S4.

GSL profile and concentrations varied across accessions within and between cabbage morphotypes. From five to nine individual GSLs were identified within all cabbages studied; seven aliphatic GSLs, namely sinigrin (SIN), gluconapin (GPN) and epi/progoitrin (PROG), Glucoibeverin (GIBVN), glucoerucin (GER), glucoiberin (GIBN) and glucoraphanin (GRPN) and two indole GSLs: glucobrassicin (GBSN) and 4-hydroxyglucobrassicin (4-HOH) (Table 1). Black kale accessions had the lowest number of identified GSLs (five), while nine GSLs were identified in red and white cabbages. GBSN and 4-HOH were the only GSLs identified in all studied accessions. Total GSLs differed significantly between accessions (*p* < 0.0001), post different cooking methods (*p* < 0.0001) and in the interaction between these two factors (*p* < 0.0001). Aliphatic GSLs were the most abundant GSLs in all accessions, making up about 95% of total GSLs. 

Cooking significantly reduced GSL concentration in all cabbage samples. GSL stability varied across the accessions and cooking methods studied. GIBN was the least stable GSL resulting in an average loss of 59% across all accessions. However, GIBN loss varied largely between accessions, with tronchuda accession TC-PCM recording a loss of up to 83%, while the loss in savoy SC-HSC was as low as 14%. The obtained results agree with those reported by Oerlemans et al. [28] and Dekker et al. [63], who report variations in GSL stability between GSLs and variations in the stability of the same GSL across different *Brassica* vegetables. In a previous study, concentrations of GIBN (aliphatic GSL) and GBSN (indole GSL) in white cabbage were found to significantly decrease during cooking due to their high potential to leach into the cooking water [64,65].

Total GSLs in steamed cabbage ranged between 16.5 µmol/g DW (BK-CNDTT) and 148.8 µmol/g DW (WD-8714). There was a significant difference in GSL concentrations of steamed cabbages across accessions and between accessions of the same cabbage morphotype. The observed differences were mostly due to the initial GSL concentration of the raw samples rather than the steaming process. In relation to the residual GSL content of cabbage samples after steaming, steamed WC-FEM had the most stable total GSL, retaining up to 97% GSL concentration, while the biggest loss of total GSL was in steamed SC-SDG, where up to 56% loss was recorded. The differences observed in GSL stability may be due to variations in leaf thickness between the accessions, which would impact the rate of heat transfer within the leaves. Thicker leaves would lead to a slower heat transfer rate within the leaves, resulting in reduced GSL degradation and better stability compared to thinner leaves. In some accessions, steaming did not affect the concentrations of some individual GSLs, e.g., SIN and PROG in WD-8714 and WC-FEM, respectively. There was a significant (*p* < 0.0001) reduction in individual and total GSL content for all samples after cooking, except for GPN, which did not differ significantly from raw to cooked samples within each accession for most of the studied accessions. The stability of individual GSLs varied greatly between accessions, within and between cabbage morphotypes. For example, after steaming, in BK samples, the loss of GRPN did not differ between the three accessions (8–10%), while in TC samples, it led to a between 44% (TC-CPDP) and <1% (TC-PCM) loss of GRPN content.

Previous studies reported no loss [25,29,35,66,67] or minimal losses [30,31,35,68] of GSL in broccoli, turnip, and cabbages after domestic processing. Xu et al. [30] reported a loss of about 15% in steamed red cabbage; however, the large sample size (3 cm cubes) may have caused lower losses in comparison to the present study, as this would have had an impact on the core temperature of the samples during cooking. Similar to the current study, Vallejo et al. [31] reported losses in some individual GSL (GRPN) and no loss in others (GIBN) after steaming for 3.5 min. In kale samples steamed for 15 min, SIN degraded more rapidly than GIBN, with more than 80% and 40% loss recorded for SIN and GIBN, respectively [33]. The minimal GSL losses reported in steamed samples were due to the low levels of leaching into cooking water compared to that normally reported under boiling conditions [23].

In microwaved samples, total GSL varied between 11.2 µmol/g DW (BK-CNDTT) and 131.6 µmol/g DW (WD-8714). Microwaving significantly affected the amount of GSLs in cabbage samples, with reductions up to 76% of GRPN in TC1 and residual total GSL varying between 50% and 93%. Microwaving led to significantly lower GSL concentrations when compared to raw cabbages. As in steamed samples, the effect of microwaving differed between accessions and individual GSLs. Some GSLs were more stable than others in certain accessions within and between cabbage morphotypes. As discussed in Section 3.1, high core temperatures (85–95 °C) of microwaved samples led to myrosinase enzyme inactivation, which could have prevented GSL hydrolysis during the microwave process and can account for the high retention of GSL concentrations in some microwaved cabbages.

There are several conflicting reports on the effect of microwaving on GSL content in *Brassica* vegetables. In a recent study, no significant difference was observed in broccoli and red cabbage samples microwaved under different time and power combinations while retaining the same final energy (1080 kJ) [35]. Song and Thornalley [29] and Xu et al. [30] also reported no significant difference in GSL concentration after microwaving green and red cabbage samples for three and five minutes, respectively. The authors stated that the stability of GSL might be due to myrosinase inactivation, and that the absence of water during microwaving prevented GSL leaching into cooking water. The large size of the shredded cabbage pieces in the two studies may also have reduced the loss of GSLs. A study on broccoli resulted in a 74% decrease in total GSL content after microwaving and was attributed to leaching in water and more intense microwave conditions (150 g broccoli to 150 g water and microwaving for 5 min at 1000 W power) [31]. However, a contrary result was observed by Verkerk and Dekker [24] and Oerlemans et al. [28], who reported an increase of up to 78% and 35%, respectively in GSL concentrations after microwaving red cabbage, though the increase was not significant in the Oerlemans et al. [28] study due to the large sample variability. The authors attributed the increase to the enhanced extractability of GSL after microwaving, which could be more of an analytical artefact than an actual increase in GSL concentration.

Stir-frying led to a significant decrease in the total and individual GSL content of cabbages. Total GSL ranged between 10.3 µmol/g DW (BK-CNDTT) and 111.7 µmol/g DW (WD-8714). There was a significant difference in GSL concentrations between accessions, within and between cabbage morphotypes. Residual total GSL varied between 28% (SC-SDG) and 81% (SC-HSC). The highest loss of aliphatic individual GSL concentration was recorded in stir-fried TC-PCM accession, where there was a decrease of between 79 to 83%. Indole GSLs, GBSN and 4-HOH were the most stable GSLs in stir-fried cabbages. The relative thermostability of individual GSLs (if under the same myrosinase level and stability) can be influenced by their chemical structure and has been reported to vary with heating temperature [28,69]. Among all the studied cooking methods, stir-frying resulted in significantly greater losses of GSL than steaming or microwaving, which agrees with previous reports. A study on the effect of different types of cooking oil on GSLs in stir-fried broccoli resulted in up to 49% losses, irrespective of the cooking oil used [70]. Xu et al. [30] also reported a 77% loss in GSL concentration after stir-frying red cabbage while there was no significant loss in GSL content when green cabbage was stir-fried for 5 min [29]. The difference in leaf structure may have influenced GSL stability in green cabbage. Green cabbage can have thicker leaves with a more uneven surface texture, which may create a microclimates around the leaf during the cooking process [29]. It was observed in the present study that green cabbage tended to have thicker leaves than other morphotypes based on visual observation. It is hypothesized that losses due to stir-frying can be attributed to substantial moisture evaporation. During stir-frying, cabbage loses moisture and GSLs are leached into the moisture, which evaporates during the cooking process. A study conducted by Adler-Nissen [56] showed that when carrot cubes were stir-fried, despite temperatures only reaching 70 °C, a high evaporation loss was observed. Another possible reason for the lower GSL amounts in stir-fried cabbages can be attributed to GSL hydrolysis by myrosinase and ESP during the cooking process. As mentioned previously, (see Section 3.1 and Section 3.2), the low core temperatures (65–70 °C) of stir-fried cabbages resulted in higher myrosinase stability of the samples when compared to steamed and microwaved cabbages. Contrary to the findings of this study, however, some recent studies showed that stir-frying preserved total and most individual GSL contents of various *Brassica* vegetables, with the authors attributing this to quick myrosinase inactivation and no leaching losses into cooking water [33,71,72].

The relative stability of individual and total aliphatic GSLs to indoles varied between accessions and cooking methods, however, generally, indole GSLs were more stable than aliphatics. Previous studies have largely reported indole GSLs to be more heat-labile under domestic cooking conditions than aliphatic GSLs [31,33,64,73,74,75]. The higher stability of indoles in the present study may be due to the type of *Brassica* and cooking methods that were investigated. The nature of the vegetable matrix and heat treatment have been shown to have an effect on the stability of individual indole GSLs. For example, in the absence of leaching and enzymatic degradation during thermal treatment, 4HOH has been reported to be more thermolabile than GBSN and neoglucobrassicin (NEO) [28,75,76]. NEO has also been found to be more thermostable than GIBVN after roasting broccoli sprouts for 15 min [75]. It is important to mention that the GSL concentrations obtained in the current study are much higher than those reported for mature cabbage in the literature [62,65,77,78,79,80]. However, up to 80 µmol/g DW [81] and 111 µmol/g DW [82] have been reported in mature rocket leaves, which is similar to the concentrations reported for most accessions in this study. We hypothesis that the reason for the high GSL accumulation in the accessions studied may be because they are gene bank accessions, which means they have not been thoroughly characterized for their phytochemical content. It is common for wild Brassicas to have much higher concentrations than cultivated varieties, and many of the high GSL genotypes that have been bred came from crosses with gene bank material, an example of which is the “*Beneforte*” broccoli [83].

In summary, WD-8707 accession had the most stable individual and total GSL, while GSLs of SC-SDG were the most thermolabile across all cooking methods, despite having one of the highest GSL concentrations in the raw sample. Different accessions of the same cabbage morphotype can vary in their GSL stability during cooking, resulting in large differences in GSL loss between species. The rate and extent of loss is dependent on the morphotype of the cabbage, sample cut size, cooking time and temperature, amount of moisture, and initial concentration of GSL [65,66]. The variation in residual GSL in the cabbages will have an impact on the amounts of GHPs produced.

### 3.5. Effect of Domestic Cooking on GHP Profile and Concentration in Cabbage Accessions

**Figure 3:**
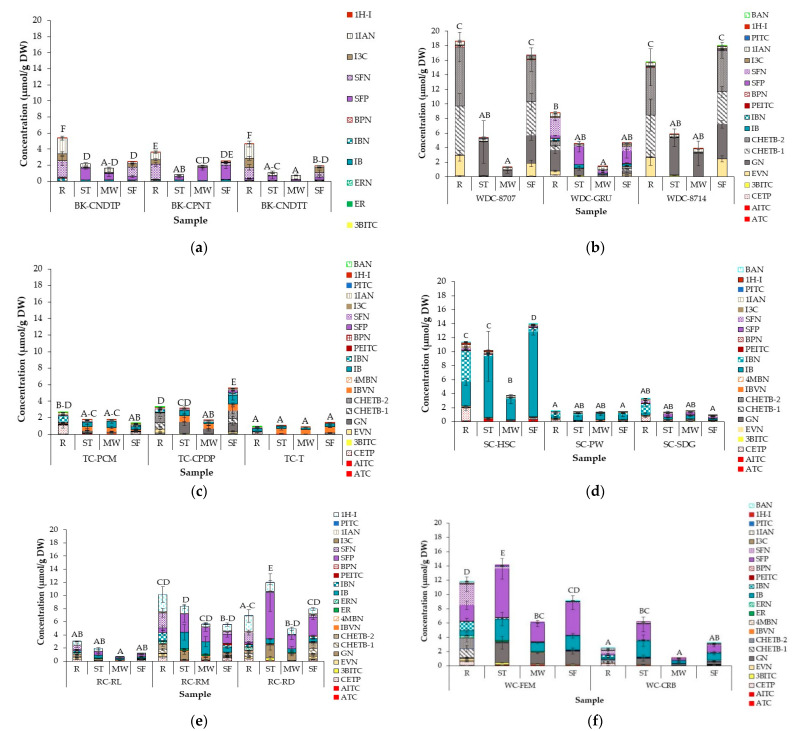
Glucosinolate hydrolysis products (GHPs) (µmol/g DW) in different accessions of (**a**) black kale; (**b**) wild cabbage (**c**) tronchuda cabbage; (**d**) Savoy cabbage; (**e**) Red cabbage and (**f**) white cabbage before and after domestic cooking. Results are expressed as sulforaphane equivalents. Error bars represent standard deviation from mean values. Letters above bars refer to differences in total GHP concentration. Letters ‘A–F’: bars not sharing a common uppercase letter differ significantly (*p* < 0.05) between accessions and cooking methods within a cabbage morphotype (i.e., within each separate graph). For significant differences between cabbage morphotypes, accessions, and cooking methods (i.e., across the separate cabbage morphotype graphs), see Supplementary Table S5. Compounds with similar colour shades are GHPs (with nitriles in pattern fill) of corresponding GSL, presented in Figure 2. Abbreviations: R = raw, ST = steamed; MW = microwaved and SF = stir-fried; BK-CPNT: cavolo palmizio; BK-CNDTT: cavolo nero di toscana o senza testa; WD-8707: wild cabbage 8707; WD-GRU: wild cabbage 7338; WD-8714: wild cabbage 8714; TC-PCM: penca mistura; TC-CPDP: penca povoa; TC-T: tronchuda; SC-HSC: hybrid savoy wirosa; SC-PW: pointed winter; SC-SDG: dark green; RC-RL: red langendijker; RC-RM: rocco marner (Hybrid); RC-RD: red Danish; WC-FEM: early market; WC-CRB: couve repolho. For abbreviations of compounds, see Table 2 (GHPs).

The profile and concentration of GHPs resulting from cooking cabbage are presented in Figure 3, with significant differences between and within the cabbage morphotypes presented in Supplementary Table S5. Twenty-three (23) different GHPs were detected as a result of GSL hydrolysis during cooking. Accession, cooking method and interaction between the two significantly (*p* < 0.0001) influenced GHP profile and concentrations. Total GHPs across all accessions and cooking methods ranged between 0.33 µmol/g DW (microwaved TC-T) and 18.66 µmol/g DW (raw WD-8707). In raw samples, GSL hydrolysis led to the production of majorly nitriles and epithionitriles. Matusheski and Jeffery [84] and Mithen et al. [85], in their studies of fresh and freeze-dried raw broccoli, found that GRPN hydrolysis primarily led to the formation of SFN rather than its ITC, SFP. In most studied accessions, raw and stir-fried cabbages had the highest total GHPs in all samples, apart from red and white cabbage accessions, where the highest total GHPs was recorded in steamed cabbages. Black kale samples had the lowest identified GHPs, which could be related to the lower number of individual GSL present in the accession.

However, some GHPs were identified where intact GSL was not detected, and this occurred across all tested accessions. In black kale and savoy cabbage accessions, 3-butenyl-ITC (3BITC) was detected in cooked samples though intact GPN was not present. A similar trend was noticed by Bell et al. [44], who found 3BITC in rocket samples, in the absence of GPN. The presence of 3BITC might be the result of SFP degradation. A study conducted on broccoli showed that standard SFP solution was degraded to 3BITC under thermal conditions [86]. PEITC and benzenepropanenitrile (BPN), hydrolysis products of gluconasturtiin, were detected in low amounts across all accessions, although intact gluconasturtiin was not detected in samples. The small amounts detected suggest that the GSL was present in low amounts in the sample and may have been hydrolysed during sample preparation, or the amounts present were below the limit of detection for the LC-MS.

Other studies have reported the presence of GHPs where their precursor, GSL, was not detected in different *Brassicas* [82,87,88]. Bell et al. [82] suggested that this discrepancies may be due to the degradation of other hydrolysis products during the analytical process, the inaccurate identification of GSLs and GHPs, very low amounts of GSLs being present in the plant, which were below the limit of detection (LOD) of the analytical method, or a yet-to-be identified mechanism by which GHPs are modified after hydrolysis. 

Cooking significantly reduced the nitriles and EPTs that were formed and increased the number of ITCs compared to raw cabbage. Goitrin (GN), iberin (IB) and SFP were the major GHPs in cooked cabbages. Of all the studied cooking methods, microwaved samples had the lowest levels of GHPs; few or no nitriles and EPTs were detected, while very low amounts of ITCs were formed. However, in most cases, more ITCs were formed in microwaved samples when compared to raw samples. The highest concentrations of ITCs were formed in steamed samples across all studied accessions, with up to 23-fold increases in ITCs, compared to those observed in raw samples (SFP in steamed RC-RD), where few or no nitriles were present. In most samples, total and individual GHPs did not significantly differ between stir-fried and raw samples, although higher numbers of ITCs were formed in stir-fried samples. The pattern of GHP formation did not differ across accessions.

Differences in the severity of cooking methods, which may have influenced residual myrosinase activity in relation to ESP activity, can account for the difference in the types and concentration of GHPs present. ESP promotes the formation of nitriles and EPTs from GSL hydrolysis instead of ITCs from myrosinase [3]. The stir-fry cooking temperature was the least severe, leading to the formation of EPTs, nitriles and ITCs, as ESP and myrosinase would have still been active in the samples. The lower amounts of GSL detected in stir-fried cabbages did not seem to affect total GHPs but might have been partly responsible for the higher amounts of nitriles formed, as GSL was hydrolysed by the ESP present in the samples during the stir-frying process. Microwave cooking was the most severe cooking method employed, which was responsible for the negligible amounts of nitriles and low amounts of ITCs. The high core temperatures during microwaving (85–95 °C) would have led to the complete denaturation of ESP and almost total myrosinase inactivation, as highlighted earlier (Section 3.1). However, the steaming temperature would have been enough to denature ESP whilst still retaining substantial myrosinase activity (see Section 3.1 and Section 3.2). The nitriles detected in both microwaved and steamed samples may have been formed with the residual ESP present during the cooking process, while the ITCs present in microwaved samples could be the result of residual myrosinase activity. In cooked broccoli, ESP was found to be denatured at temperatures above 50 °C, with a corresponding reduction in SFN production [3]. Rungapamestry et al. [25], in their study of SIN hydrolysis products in cooked cabbage, found that microwaving for 120 secs resulted in a reduction in nitriles, allyl cyanide and CEP (about 87%), with an increase in AITC formation (about 88%). The authors found that steaming cabbages for seven minutes resulted in an increase in AITC of up to 578%. The authors also found that AITC was formed in cabbages with no residual myrosinase activity and attributed this to formation during the hydrolysis and cooking process, which may have been bound to the cell membranes but released during processing. In a recent study, the steaming and stir-frying of broccolini and kale for 15 min resulted in significantly lower amounts of SFP and IB, respectively, when compared to the uncooked sample. The lower concentrations reported were probably due to the longer cooking time used in the study, which would have resulted in total myrosinase inactivation, preventing the conversion of GSLs to GHPs, although myrosinase activity was not measured in the study [33]. In another study, steaming broccoli resulted in increased SFP content after 5 min with a decrease observed beyond that, while microwaving broccoli and red cabbage for a minute led to a 5-fold increase in SFP with a decline reported beyond this time [35]. This suggests that there is an optimum cooking time to achieve maximum ITC formation, beyond which beneficial ITCs are lost. The low conversion of GSLs to their hydrolysis products in some of the studied samples is underscored. The results show that GHP recovery reduced with increases in the severity of the cooking procedure, with much lower concentrations (about 1% in microwaved TC-T) observed in microwaved samples, which was the most severe treatment employed. The low recovery of hydrolysis products observed in the microwaved samples is not necessarily a surprise, given that most of the ESP and myrosinase enzyme in the samples were already inactivated as a result of the cooking temperature (88–95 °C), as discussed in Section 3.1 and Section 3.2 (see Figure 1 and Supplementary Table S3), which suggests that most of the GSL present in the sample remained unhydrolysed. However, the GSL concentration of the hydrolysed sample was not measured in the current study. In raw accessions with a low GHP content, we hypothesise that this may be due to the environmental responses of the plant, which, unfortunately, are not very well understood in the context of myrosinase activity and GSL hydrolysis. The low conversion of GSLs to GHPs has also been reported in some other *Brassica* species [44,81].

The results obtained in this study are similar to those observed by several authors during the thermal processing of *Brassica* vegetables [25,29,32,35,55,89]. This study adds to the findings of previous researchers; however, the study is particularly conclusive as it demonstrates similar findings across cabbage morphotypes and accessions. To improve the health benefits derived from cabbage consumption, this paper concludes that steaming is the optimum preparation method, due to the resulting increase in the ITCs formed.

### 3.6. Principal Component Analysis (PCA) and Multifactor Analysis (MFA) of GSLs and GHPs in Raw and Cooked Cabbage

**Figure 4:**
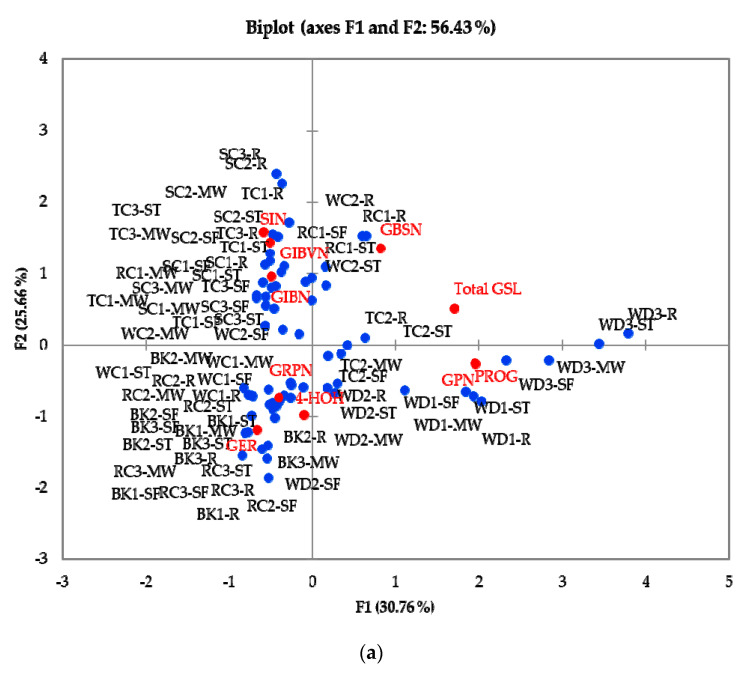

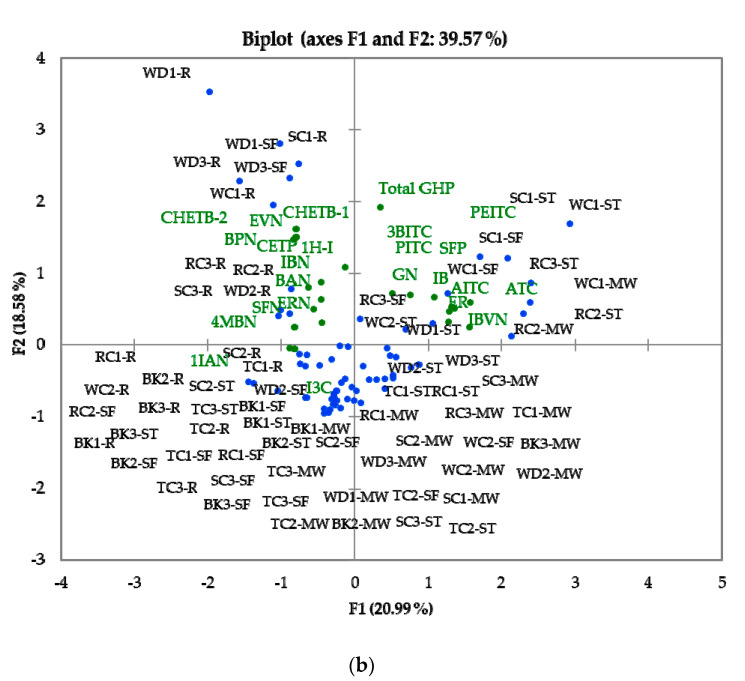
(**a**) PCA plot for tested samples and their relative distributions in relation to GSL concentrations. (**b**) PCA plot for tested samples and their relative distributions in relation to GHP concentrations. Abbreviations: R = raw, ST = steamed; MW = microwaved; SF = stir-fried; BK1: cavolo nero di toscana o senza palla (BK-CNDTP); BK2: cavolo palmizio (BK-CPNT); BK3: cavolo nero di toscana o senza testa (BK-CNDTT); WD1: wild cabbage 8707 (WD-8707); WD2: wild cabbage 7338 (WD-GRU); WD3: wild cabbage 8714 (WD-8714); TC1: penca mistura (TC-PCM); TC2: penca povoa (TC-CPDP); TC3 tronchuda (TC-T); SC1: hybrid savoy wirosa (SC-HSC); SC2: pointed winter (SC-PW); SC3: dark green (SC-SDG); RC1: red langendijker (RC-RL); RC2: rocco marner (Hybrid) (RC-RM); RC3: red Danish (RC-RD); WC1: early market (WC-FEM); WC2: couve repolho (WC-CRB). Red coloured compounds = GSLs; Green coloured compounds = GHPs; Blue dots = Samples. For full names of compounds, see Table 1 and Table 2.

To differentiate samples based on their GSLs and GHPs content, PCA analysis was conducted, as shown in Figure 4. Figure 4a shows the biplot for GSL distribution in samples, where dimensions 1 and 2 account for 56.4% of the observed variation. The plot shows TC2 (TC-CPDP), and wild cabbage accessions were characterized by high PROG and GPN contents, while black kale and most red cabbages, except for RC1 (RC-RL), had a higher tendency to accumulate GRPN and GER. Savoy cabbages, RC1 (RC-RL), TC1 (TC-PCM) and TC3 (TC-T) correlated positively with one another and were characterized by the amounts of SIN, GIBVN and GIBN they accumulated. Samples were separated based on cabbage morphotype and accession rather than cooking methods, suggesting that cabbage accession had a higher influence on GSL concentration than the tested cooking methods. However, the PCA biplot for GHPs (Figure 4b) shows differentiations in samples based on cooking. F1 and F2 explain only 39.6% of the variations; however, other dimensions did not provide any new information. Steamed and stir-fried cabbages correlated positively with ITCs, while nitriles mostly correlated with raw cabbages. There was no correlation observed in microwaved samples with GHPs, due to the low amounts of nitriles and ITCs present in the samples. Samples were separated based on their GHP profile and concentrations.

**Figure 5:**
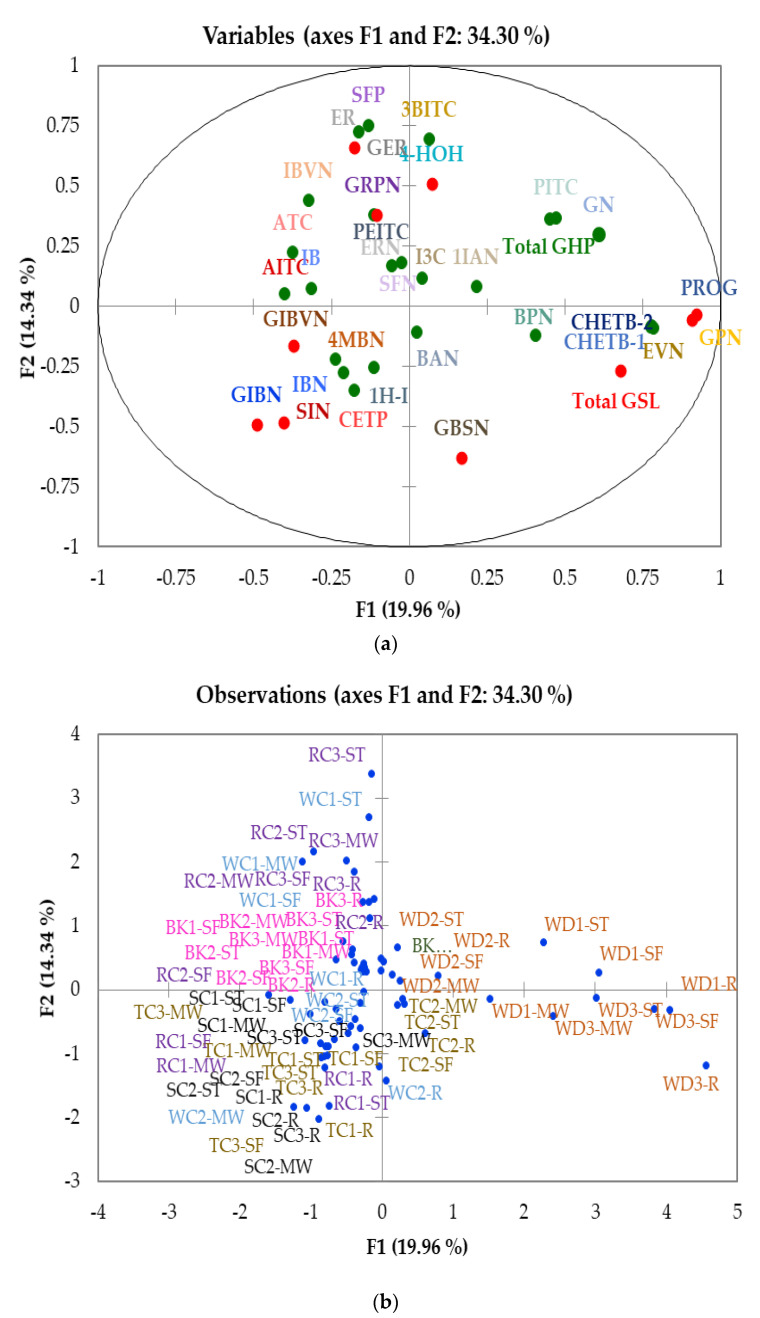
MFA map of glucosinolates and glucosinolate hydrolysis products (**a**) distribution of variables and (**b**) sample distribution. Abbreviations: R = raw, ST = steamed; MW = microwaved; SF = stir-fried; BK1: cavolo nero di toscana o senza palla (BK-CNDTP); BK2: cavolo palmizio (BK-CPNT); BK3: cavolo nero di toscana o senza testa (BK-CNDTT); WD1: wild cabbage 8707 (WD-8707); WD2: wild cabbage 7338 (WD-GRU); WD3: wild cabbage 8714 (WD-8714); TC1: penca mistura (TC-PCM); TC2: penca povoa (TC-CPDP); TC3 tronchuda (TC-T); SC1: hybrid savoy wirosa (SC-HSC); SC2: pointed winter (SC-PW); SC3: dark green (SC-SDG); RC1: red langendijker (RC-RL); RC2: rocco marner (Hybrid) (RC-RM); RC3: red Danish (RC-RD); WC1: early market (WC-FEM); WC2: couve repolho (WC-CRB). Colour codes: Pink = Black kale; Brown = Wild cabbage; Dijon yellow = Tronchuda cabbage; Black = Savoy cabbage; Purple = Red cabbage; Blue = White cabbage. Compounds with different shades of the same colour in Figure 5a refer to the GSL and corresponding GHPs. For compound codes on plot, refer to Table 1 and Table 2.

To better understand the results, MFA was performed on the accessions in relation to their GSL and GHP concentrations, as shown in Figure 5. Dimensions 1 and 2 (F1 and F2) represent only 34.3% of the variations, but other dimensions did not provide additional information. The observed results are similar to those observed in the biplot of GSL. Samples were separated in the same pattern as GSLs, based on cabbage morphotype and accession rather than cooking method. Individual GSLs correlated with their corresponding GHPs. The results observed from the MFA analysis confirm the results obtained from the PCA analysis, confirming the robustness of the findings. The results show that cooking has a greater effect on GHPs than GSLs but, when combined, samples were differentiated on their GSL content and the type of GHP present.

This study is not without its limitations. Although precautions were followed to ensure accuracy during sample preparation, the size of cut cabbages, stirring during stir-frying and general reproducibility of the cooking processes across all samples may have slightly differed. In addition, some very volatile GHPs may have been lost during the cooking and analytical processes due to the long extraction method used, which may have affected the results. However, given that standard measures were employed to limit variations due to the above, it is unlikely that possible variations within the samples could have had a significant influence on the results, as only small variations were observed in the biological and technical replicates.

## 4. Conclusions

The results of this study confirm that domestic cooking has an effect on myrosinase stability, GSL concentration and GHP profiles, and concentration. Domestic cooking resulted in a significant loss of myrosinase activity, with stir-frying having the highest residual activity compared to the other two cooking methods that were investigated. Microwave cooking was the most severe heat treatment, resulting in the highest loss of myrosinase activity, reaching up to 99% in some cases. The study showed that mild cooking prevents the complete inactivation of myrosinase enzyme. Myrosinase enzyme stability differed significantly between cabbage accessions and morphotypes. Black kale myrosinase was the most stable after stir-frying, while red cabbage accessions were most stable after steaming and microwaving. No correlation was found between myrosinase activity and stability, as the accessions with the highest myrosinase activity did not have the most stable myrosinase after domestic processing.

Cooking led to a reduction in GSL concentrations compared to raw cabbage, with stir-frying leading to the greatest loss compared to the other two cooking methods and mild steaming enabling the greatest retention of GSL compounds. Considering that cabbages are usually consumed cooked, it important for breeders to work alongside nutritionists to select accessions with more thermally stable GSL and myrosinase for breeding, to ensure that the health benefits from cabbage consumption are not lost. The study found a relationship between cabbage core temperature during cooking, myrosinase stability and final GHPs profile. GHPs of raw cabbages were mainly nitriles and EPTs, probably due to the presence of active ESP in the samples. Cooking led to a reduction in the number of nitriles and EPTs formed, with levels differing between cooking methods. Optimal cooking conditions led to the degradation of ESP but retention of active myrosinase. Microwaving resulted in significantly lower amounts of nitriles, EPT and ITC formed, while steaming cabbages led to the production of significantly higher amounts of ITCs. However, the study showed that low residual myrosinase activity can still result in ITC formation.

The study concludes that consumption of raw or severely heat-treated cabbage can reduce possible health benefits, while mild cooking of cabbages, such as mild steaming, maximises beneficial isothiocyanate formation. This was especially true for IB and SFP in the studied cabbages and could provide information to guide consumers on how to improve the possible health benefits derived from cabbage consumption.

## Figures and Tables

**Table 1:** Intact glucosinolates identified in cabbage accessions analysed by LC-MS.

Common Name	Chemical Name	Abbreviation	Mass Parent Ion	MS^2^ Spectrum Ion (Base Ion in Bold)	Reference
sinigrin	2-propenyl(allyl)GSL	SIN	358	278, 275, **259**, 227, 195, 180, 162	[45,46]
gluconapin	3-butenyl GSL	GPN	372	292, 275, **259**, 195, 194, 176	[45,47]
epi/progoitrin	(R, S)-2-hydroxy-3-butenyl GSL	PROG	388	332, 308, 301, 275, **259**, 210, 195, 146, 136	[45,46,47]
glucoiberverin	3-(methylthio)propyl GSL	GIBVN	406	326, 275, **259**, 288, 228,195	[43,45,46]
glucoerucin	4-(methylthio)butyl GSL	GER	420	340, 291, 275, **259**, 227, 195, 178, 163	[43,45,46]
glucoiberin	3-(methylsulfinyl)propyl GSL	GIBN	422	407, **358**, 259	[45,46,47]
glucoraphanin	4-(methylsulfinyl)butyl GSL	GRPN	436	422, **372**, 291, 259, 194	[43,45,46]
glucobrassicin	3-indolylmethyl GSL	GBSN	447	275, **259**, 251, 205	[45,46,47]
4-hydroxyglucobrassicin	4-hydroxy-3-indolylmethyl GSL	4-HOH	463	383, **285**, 267, 259, 240, 195	[45,46,47]

Key: GSL = glucosinolate.

**Table 2:** Glucosinolate hydrolysis products identified in cabbage accessions analysed by GC-MS.

Precursor Glucosinolate	Glucosinolate Hydrolysis Product		Abbreviation	LRI ^a^	ID ^b^	MS^2^ Spectrum Ion (Base Ion in Bold)	Reference
	Common Name	Chemical Name					
sinigrin	allyl thiocyanate	2-propenyl thiocyanate	ATC	871	B	99, 72, 45, 44, **41**, 39	[48]
	allyl-ITC	2-propenyl isothiocyanate	AITC	884	B	**99**, 72, 71, 45, 41, 39	[48,49]
	1-cyano-2,3-epithiopropane	3,4-epithiobutane nitrile	CETP	1004	B	**99**, 72, 66, 59, 45, 41, 39	[48]
gluconapin	3-butenyl-ITC	1-butene, 4-isothiocyanate	3BITC	983	B	113, 85, **72**, 64, 55, 46, 45, 41	[48,49,50]
	4,5-epithiovaleronitrile	1-cyano-3,4-epithiobutane	EVN	1121	B	**113**, 86, 80, 73, 60, 45	[50]
progoitrin	goitrin	5-vinyloxazolidin-2-thione	GN	1545	B	**129**, 86, 85, 68, 57, 45, 43, 41, 39	[51]
	1-cyano-2-hydroxy-3,4-epit-hiobutane isomer 1	2-hydroxy-3,4-epithiobutylcyanide diastereomer-1	CHETB-1	1225	B	**129**, 111, 89, 84, 68, 61, 58, 55, 45	
	1-cyano-2-hydroxy-3,4-epit-hiobutane isomer 2	2-hydroxy-3,4-epithiobutylcyanide diastereomer-2	CHETB-2	1245	B	**129**, 111, 89, 84, 68, 61, 58, 55, 45	
glucoiberverin	iberverin	3-methylthiopropyl-ITC	IBVN	1307	B	147, **101**, 86, 73, 72, 61, 47, 46, 41	[48]
	4-methylthiobutyl nitrile	4-methylthio butanenitrile	4MBN	1085	B	115, 74, 68, **61**, 54, 47, 41	
glucoerucin	erucin	4-(methylthio)-butyl-ITC	ER	1427	B	161, 146, **115**, 85, 72, 61, 55	[48,49]
	erucin nitrile	1-cyano-4-(methylthio) butane	ERN	1200	B	129, 87, 82, **61**, 55, 48, 41, 47	
glucoiberin	iberin	3-methylsulfinylpropyl-ITC	IB	1617	B	163, 130, 116, 102, 100, 86, **72**, 63, 61,41	[48]
	iberin nitrile	4-methylsulfinylbutanenitrile	IBN	1384	B	**131**, 78, 64, 47, 41	
gluconasturtin	2-phenylethyl-ITC	2-isothiocyanatoethyl benzene	PEITC	1458	B	163, 105, **91**, 65, 51, 40	[48]
	benzenepropanenitrile	2-phenylethyl cyanide	BPN	1238	B	131, **91**, 85, 65, 63, 57, 44, 51	[50]
glucoraphanin	sulforaphane	4-methylsulfinylbutyl-ITC	SFP	1757	A	160, 114, 85, **72**, 64, 63, 61, 55. 41, 39	[44,49]
	sulforaphane nitrile	5-(methylsulfinyl) pentanenitrile	SFN	1526	B	145, 128, 82, 64, **55**, 41	
glucobrassiccin	indole-3-carbinol	1H-indole-3-methanol	I3C	1801	B	**144**, 145, 116, 108, 89	[51]
	indoleacetonitrile	1H-indole-3-acetonitrile	1IAN	1796	B	**155**, 145, 144, 130, 116, 89, 101, 63	[52]
pentyl glucosinolate	pentyl-ITC	1-isothiocyanato-pentane	PITC	1165	B	129, 114, 101, 96, 72, 55, **43**, **41**, 39	[53]
indole	1H-indole	Indole (8CI)	1H-I	1290	B	**117**, 90, 89, 63, 58	[54]
glucotropaeolin	benzeneacetonitrile	2-phenylacetonitrile	BAN	1137	A	**117**, 90, 89, 77, 63, 51	

Key: ITC- isothiocyanate. ^a^ Linear retention index on a HP-5MS non-polar column. ^b^ A, mass spectrum and LRI agree with those of authentic compound; B, mass spectrum agrees with reference spectrum in the NIST/EPA/NIH mass spectra database and those in the literature.

**Table 3:** Protein content ((mg/g ± SD) DW) and specific activity ((U/mg soluble protein ± SD) DW) of cabbage accessions before and after domestic processing.

Cabbage Morphotype ^a^/Accession	Protein Content (mg/g ± SD) DW				Specific Activity (U/mg Soluble Protein ± SD) DW			
	Raw	Steamed	Microwaved	Stir-Fried	Raw	Steamed	Microwaved	Stir-Fried
**Black Kale**								
BK-CNDTP	33.7 ± 0.6 ^no, E^	11.0 ± 0.3 ^ab, A^	11.2 ± 0.4 ^ab, A^	29.0 ± 0.7 ^kl, D^	1.3 ± 0.2 ^d–k, D^	0.2 ± 0.0 ^a, A^	0.2 ± 0.0 ^a, A^	1.0 ± 0.1 ^a–j, C^
BK-CPNT	35.4 ± 1.0 ^op, EF^	11.7 ± 0.6 ^b, A^	11.9 ± 1.4 ^b, A^	21.6 ± 1.9 ^hi, B^	0.9 ± 0.1 ^a–i, BC^	0.3 ± 0.1 ^abc, A^	0.3 ± 0.1 ^abc, A^	0.7 ± 0.0 ^a–h, B^
BK-CNDTT	36.7 ± 0.7 ^p, F^	12.7 ± 0.1 ^bc, A^	12.5 ± 0.1 ^bc, A^	24.9 ± 1.6 ^j, C^	1.0 ± 0.0 ^a–j, C^	0.2 ± 0.1 ^a, A^	0.3 ± 0.1 ^ab, A^	0.8 ± 0.1 ^a–h, BC^
**Wild**								
WD-8707	31.4 ± 0.1.2 ^lmn, E^	11.1 ± 0.1 ^ab, A^	10.9 ± 0.1 ^ab, A^	19.1 ± 0.4 ^fgh, C^	1.6 ± 0.1 ^g–l, C^	0.2 ± 0.1 ^a A^	0.2 ± 0.0 ^a, A^	1.1 ± 0.1 ^a–j, B^
WD-GRU	29.9 ± 0.6 ^kl, D^	10.7 ± 0.4 ^ab, A^	10.6 ± 0.1 ^ab, A^	18.1 ± 1.1 ^efg, C^	1.7 ± 0.2 ^h–l, C^	0.3 ± 0.1 ^a–c A^	0.2 ± 0.0 ^a, A^	1.2 ± 0.2 ^c–k, B^
WD-8714	30.6 ± 0.8 ^lm, DE^	10.9 ± 0.1 ^ab, A^	11.0 ± 0.2 ^ab, A^	16.9 ± 0.5 ^def, B^	2.4 ± 0.2 ^l, D^	0.2 ± 0.0 ^a A^	0.2 ± 0.0 ^a, A^	1.7 ± 0.1 ^h–l, C^
**Tronchuda**								
TC-PCM	33.6 ± 0.2 ^no, F^	11.1 ± 0.3 ^ab, A^	11.1 ± 0.1 ^ab, A^	19.9 ± 1.47 ^gh, D^	1.2 ± 0.1 ^b–k, D^	0.3 ± 0.1 ^a–c A^	0.3 ± 0.1 ^a–c, A^	0.7 ± 0.1 ^a–g, C^
TC-CPDP	27.8 ± 0.6 ^k, E^	11.0 ± 0.3 ^ab, A^	11.0 ± 0.3 ^ab, A^	18.1 ± 0.8 ^efg, C^	2.4 ± 0.3 ^l, E^	0.4 ± 0.2 ^a–e ABC^	0.4 ± 0.1 ^a–e, AB^	1.4 ± 0.2 ^e–k, D^
TC-T	33.1 ± 0.8 ^mno, F^	10.9 ± 0.2 ^ab, A^	10.8 ± 0.2 ^ab, A^	15.7 ± 0.9 ^de, B^	1.4 ± 0.1 ^f–k, D^	0.3 ± 0.1 ^abc A^	0.3 ± 0.1 ^abc, A^	0.6 ± 0.1 ^a–f, BC^
**Savoy**								
SC-HSC	24.6 ± 1.43 ^j, D^	10.7 ± 0.4 ^ab, AB^	10.6 ± 0.3 ^ab, AB^	12.0 ± 1.1 ^b, B^	4.7 ± 0.3 ^n, B^	0.4 ± 0.1 ^a–d A^	0.2 ± 0.0 ^ab, A^	1.8 ± 0.2 ^i–l, A^
SC-PW	24.3 ± 0.3 ^j, D^	12.0 ± 1.2 ^b, B^	10.1 ± 0.2 ^b, AB^	14.8 ± 0.4 ^cd, C^	6.4 ± 0.5 ^o, B^	0.2 ± 0.0 ^a A^	0.2 ± 0.0 ^a, A^	0.3 ± 0.0 ^abc, A^
SC-SDG	24.4 ± 0.5 ^j, D^	10.3 ± 0.4 ^ab, AB^	8.9 ± 0.2 ^a, A^	11.4 ± 0.3 ^ab, AB^	5.8 ± 0.7 ^o, B^	0.3 ± 0.1 ^abc A^	1.5 ± 0.1 ^f–l, A^	1.1 ± 0.1 ^a–j, A^
**Red**								
RC-RL	33.6 ± 0.6 ^no, E^	11.0 ± 0.3 ^ab, A^	11.2 ± 0.4 ^ab, A^	29.0 ± 0.7 ^kl, D^	0.9 ± 0.1 ^a–j, D^	0.4 ± 0.1 ^a–d A^	0.4 ± 0.1 ^a–d, A^	0.5 ± 0.1 ^a–f, ABC^
RC-RM	35.4 ± 1.0 ^op, EF^	11.7 ± 0.6 ^b, A^	11.9 ± 1.4 ^b, A^	21.6 ± 1.9 ^hi, B^	1.5 ± 0.3 ^g–l, E^	0.7 ± 0.1 ^a–g BCD^	0.4 ± 0.1 ^a–e, AB^	0.9 ± 0.1 ^a–i, D^
RC-RD	36.7 ± 0.7 ^p, F^	12.7 ± 0.1 ^bc, A^	12.5 ± 0.1 ^bc, A^	24.9 ± 3.9 ^j, C^	1.9 ± 0.1 ^jkl, F^	0.2 ± 0.1 ^a A^	0.3 ± 0.1 ^ab, A^	0.7 ± 0.1 ^a–h, CD^
**White**								
WC-FEM	21.3 ± 0.4 ^hi, C^	10.1 ± 0.3 ^ab, A^	10.1 ± 0.1 ^ab, A^	10.9 ± 0.2 ^ab, A^	3.4 ± 0.2 ^m, E^	0.3 ± 0.1 ^abc, A^	0.4 ± 0.1 ^a–d, AB^	0.6 ± 0.2 ^a–f, BC^
WC-CRB	23.0 ± 1.2 ^ij, D^	10.2 ± 0.1 ^ab, A^	10.2 ± 0.1 ^ab, A^	12.1 ± 0.7 ^b, B^	2.1 ± 0.1 ^kl, D^	0.3 ± 0.1 ^abc, A^	0.2 ± 0.1 ^ab, A^	0.7 ± 0.2 ^a–h, C^

^a^ Names in bold refer to cabbage morphotype. Values are means of three processing replicates and two technical replicates (*n* = 6 ± SD). SD: standard deviation from mean. Letters “A–F”: mean values not sharing a common uppercase letter differ significantly (*p* < 0.05) between accessions and treatments within a cabbage type for each parameter (i.e., protein content and specific activity). Letters “a–p”: mean values not sharing a common lowercase letter differ significantly (*p* < 0.05) between cabbage types, accessions, and treatments for each parameter (i.e., protein content and specific activity). Key: BK-CNDTP: cavolo nero di toscana o senza palla BK-CPNT: cavolo palmizio; BK-CNDTT: cavolo nero di toscana o senza testa; WD-8707: wild cabbage 8707; WD-GRU: wild cabbage 7338; WD-8714: wild cabbage 8714; TC-PCM: penca mistura; TC-CPDP: penca povoa; TC-T: tronchuda; SC-HSC: hybrid savoy wirosa; SC-PW: pointed winter; SC-SDG: dark green; RC-RL: red langendijker; RC-RM: rocco marner (Hybrid); RC-RD: red Danish; WC-FEM: early market; WC-CRB: couve repolho.

## Data Availability

The data presented in this study are available on request from the corresponding author.

## Supplementary Materials

The following are available online at https://www.mdpi.com/article/10.3390/foods10122908/s1, Figure S1: Examples of GC-MS chromatograms for raw and cooked samples for each morphotype of cabbage studied (a) black kale; (b) wild cabbage; (c) tronchuda cabbage; (d) savoy cabbage; (e) red cabbage and (f) white cabbage. Table S1: Origin, botanical and common names of planted cabbage accessions. Table S2. Consumption intent and cooking time scores from preliminary consumer study. Table S3. Relative activity (A/A0 ± SD) of myrosinase enzyme after domestic cooking of cabbage. Table S4: Glucosinolate concentration of raw and cooked cabbage (µmol/g DW). Table S5: Glucosinolate hydrolysis products concentration in raw and cooked cabbage (µmol/g DW sulforaphane equivalent).
