# Supplementary material for: The Impact of Domestic Cooking Methods on Myrosinase Stability, Glucosinolates and Their Hydrolysis Products in Different Cabbage (Brassica oleracea) Accessions
Source: Foods. 2021 Nov 24;10(12):2908. doi: 10.3390/foods10122908 (PMC8700523; doi:10.3390/foods10122908)
Supplement: Supplementary file 1 [file foods-10-02908-s001.zip › Supplementary data 1_paper 2.pdf]

Supplementary data 1

Table S1: Origin, botanical and common names of cabbage accessions planted

Genus/Type	Accession name	Accession code	Common name	Origin	Head formation
**Black kale**					
Brassica oleracea var. *acephala*	Cavolo nero di toscana o senza palla (BK-CNDTP)	BK1	Fodder black kale	Italy	Open leaf
Brassica oleracea var. *acephala*	Cavolo palmizio (BK-CPNT)	BK2	Black kale	Italy	Open leaf
Brassica oleracea var. *acephala*	Cavolo nero di toscana o senza testa (BK-CNDTT)	BK3	Fodder black kale	Italy	Open leaf
**Wild**					
Brassica oleracea	Wild cabbage (WD-8707)	WD1	Wild cabbage	Great Britain	Open leaf
Brassica oleracea	Wild cabbage (WD-GRU)	WD2	Wild cabbage	New Zealand	Open leaf
Brassica oleracea	Wild cabbage (WD-8714)	WD3	Wild cabbage	Great Britain	Open leaf
**Tronchuda**					
Brassica oleracea var. *tronchuda*	Penca mistura (TC-PCM)	TC1	Tronchuda cabbage	Portugal	Open leaf
Brassica oleracea var. *tronchuda*	Penca povoa (TC-CPDP)	TC2	Tronchuda cabbage	Portugal	Open leaf
Brassica oleracea var. *tronchuda*	Tronchuda (TC-T)	TC3	Tronchuda cabbage	Portugal	Open leaf
**Savoy**					
Brassica oleracea var. *capitata*	Hybrid savoy wirosa cabbage (SC-HSC)	SC1	Hybrid savoy cabbage	Great Britain	Closed heart
Brassica oleracea var. *capitata*	Pointed winter (SC-PW)	SC2	Savoy cabbage	Great Britain	Closed heart
Brassica oleracea var. *capitata*	Dark green (SC-SDG)	SC3	Savoy cabbage	Italy	Closed heart
**Red**					
Brassica oleracea var. *capitata*	Red langendijker (RC-RL)	RC1	Red cabbage	Great Britain	Closed heart
Brassica oleracea var. *capitata*	Rocco marner (Hybrid) (RC-RM)	RC2	Hybrid red cabbage	Great Britain	Closed heart
Brassica oleracea var. *capitata*	Red Danish (RC-RD)	RC3	Red cabbage	Netherlands	Closed heart
**White**					
Brassica oleracea var. *capitata*	Early market (WC-FEM)	WC1	White spring cabbage	Great Britain	Closed heart
Brassica oleracea var. *capitata*	Couve repolho (WC-CRB)	WC2	White cabbage	Portugal	Closed heart
Brassica oleracea var. *capitata*	De louviers (WC-DLI)	WC3	Hybrid white cabbage	Great Britain	Closed heart

Table S2. Consumption intent and cooking time scores from preliminary consumer study.

	[1] Cooking method and time (secs)					
	**Steamed**		**Stir-fried**			**Microwaved**
**Sample / Attribute**	120	180	90	120	150	180
**Closed heart cabbage (Red cabbage)**						
Cooking standard [2]	3.3	3.4	2.8	3.0	2.8	3.8
Consumption intent [3]	3.3	3.2	3.6	3.5	3.5	2.9
**Open leaf cabbaged (Black kale)**						
Cooking standard [2]	2.7	2.9	2.5	2.7	2.7	2.7
Consumption intent [3]	2.6	2.9	2.5[ab]	2.1[a]	2.6[ab]	2.3

[1] Means values from 60 consumers; mean values with different superscripts in the same row within the same cooking condition and cabbage type are significantly different at $p < 0.0001$. [2] Means measured on a Just-About-Right (JAR) scale (where 1: not cooked enough,3: JAR and 5: much too overcooked). [3] Mean values measured on a 5-point scale (where 1: definitely would not eat; 3: may or may not eat; 5: definitely would eat).

Table S3. Relative activity (A/A0±SD) of myrosinase enzyme after domestic cooking of cabbage.

Cabbage Type/Accession	Relative activity (A/A_0)		
	Treatments		
	Steamed	Microwaved	Stir-fried
**Black kale**			
BK-CNDTP	0.05±0.01$^{ab\ A}$	0.05±0.01$^{ab\ A}$	0.65±0.01$^{l\ G}$
BK-CPNT	0.11±0.01$^{a\text{-}e\ D}$	0.11±0.05$^{a\text{-}e\ D}$	0.52±0.11$^{jk\ E}$
BK-CNDTT	0.08±0.05$^{a\text{-}e\ B}$	0.09±0.05$^{a\text{-}e\ C}$	0.56±0.13$^{kl\ F}$
**Wild**			
WD-8707	0.05±0.02$^{ab\ C}$	0.05±<0.01$^{ab\ C}$	0.41±0.03$^{hij\ F}$
WD-GRU	0.08±0.03$^{a\text{-}d\ D}$	0.05±0.01$^{ab\ B}$	0.46±0.12$^{h\text{-}k\ G}$
WD-8714	0.03±<0.01$^{ab\ A}$	0.03±<0.01$^{ab\ A}$	0.38±0.03$^{hi\ E}$
**Tronchuda**			
TC-PCM	0.08±0.03$^{a\text{-}e\ D}$	0.08±0.03$^{a\text{-}e\ D}$	0.34±0.06$^{gh\ F}$
TC-CPDP	0.08±0.03$^{a\text{-}d\ C}$	0.07±0.02$^{a\text{-}d\ B}$	0.38±0.05$^{ghi\ G}$
TC-T	0.07±0.03$^{a\text{-}d\ C}$	0.06±0.02$^{a\text{-}d\ A}$	0.20±0.05$^{ef\ E}$
**Savoy**			
SC-HSC	0.04±0.01$^{ab\ E}$	0.02±0.01$^{a\ C}$	0.19±0.03$^{def\ G}$
SC-PW	0.02±0.01$^{a\ B}$	0.01±<0.01$^{a\ A}$	0.03±<0.01$^{ab\ D}$
SC-SDG	0.02±0.01$^{a\ C}$	0.02±0.01$^{a\ C}$	0.09±0.01$^{a\text{-}e\ F}$
**Red**			
RC-RL	0.13±0.04$^{a\text{-}e\ D}$	0.13±0.04$^{a\text{-}e\ D}$	0.49±0.18$^{ijk\ H}$
RC-RM	0.15±0.03$^{b\text{-}f\ E}$	0.10±0.04$^{a\text{-}e\ C}$	0.37±0.08$^{ghi\ G}$
RC-RD	0.04±0.01$^{ab\ A}$	0.05±0.02$^{ab\ B}$	0.26±0.02$^{fg\ F}$
**White**			
WC-FEM	0.04±0.02$^{ab\ A}$	0.05±0.02$^{ab\ B}$	0.09±0.02$^{a\text{-}e\ D}$
WC-CRB	0.06±0.03$^{abc\ C}$	0.05±0.02$^{ab\ B}$	0.18±0.03$^{c\text{-}f\ E}$

Values are means of three processing replicates (each replicate comprising of 4-5 cabbage heads) and two technical replicates (n=6±SD). SD: standard deviation from mean. Letters "ABC": mean values not sharing a common uppercase letter differ significantly ($p < 0.05$) between accessions and treatments within a cabbage morphotype. Letters "abc": mean values not sharing a common lowercase letter differ significantly ($p < 0.05$) between cabbage morphotypes, accessions, and treatments. A/A0 = residual activity, defined as the ratio of myrosinase activity of processed (cooked) cabbage to unprocessed (raw) cabbage. Key: BK-CNDTP: Cavolo nero di toscana o senza palla; BK-CPNT: Cavolo palmizio; BK-CNDTT: Cavolo nero di toscana o senza testa; WD-8707: Wild cabbage 8707; WD-GRU: Wild cabbage 7338; WD-8714: Wild cabbage 8714; TC-PCM: Penca mistura; TC-CPDP: Penca povoa; TC-T: Tronchuda; SC-HSC: Hybrid savoy wirosa; SC-PW: Pointed winter; SC-SDG: Dark green; RC-RL: red langendijker; RC-RM: Rocco marner (Hybrid); RC-RD: Red Danish; WC-FEM: Early market; WC-CRB: Couve repolho; WC-DLI: De louviers.

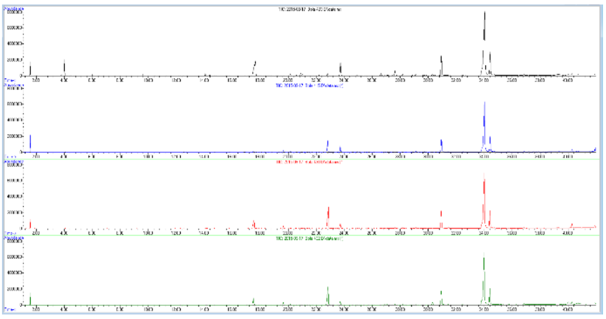

(**a**) Black kale

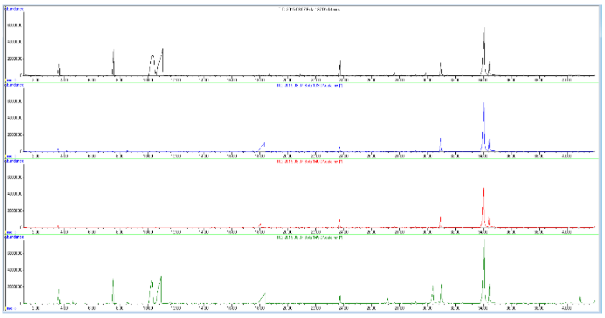

(**b**) Wild cabbage

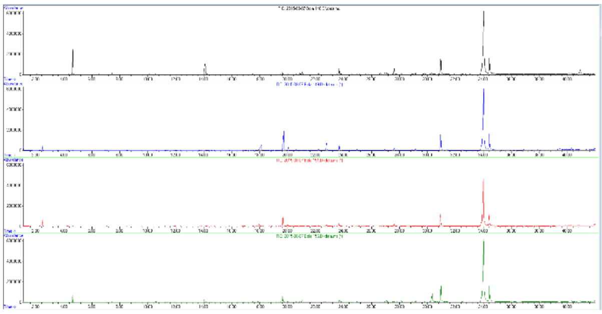

(**c**) Tronchuda cabbage

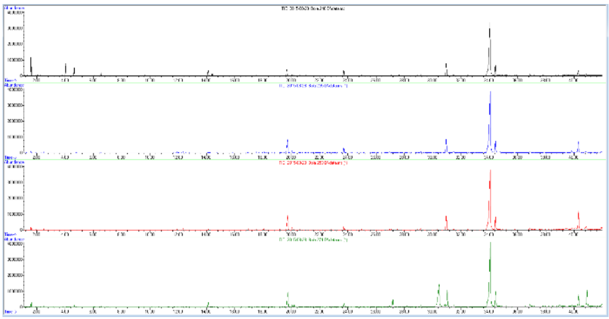

(**d**) Savoy cabbage

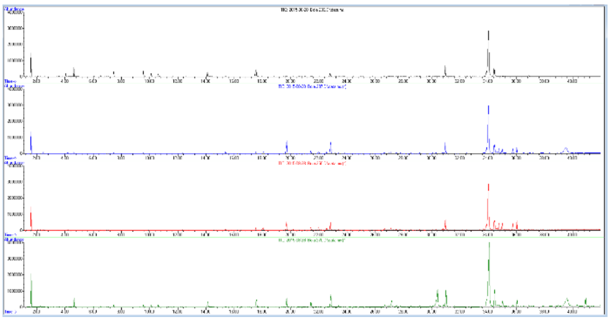

(**e**) Red cabbage

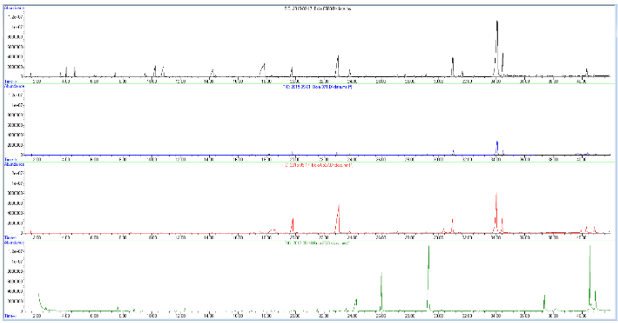

(**f**) White cabbage

Figure S1. Examples of GC-MS chromatograms for raw and cooked samples for each morphotype of cabbage studied (**a**) Black kale; (**b**) Wild cabbage; (**c**) Tronchuda cabbage; (**d**) Savoy cabbage; (**e**) Red cabbage and (**f**) White cabbage. Chromatogram colour keys: Black – Raw; Blue – steamed; Red – Microwaved and Green – stir-fried.
